# Evaluation of end-of-life vehicle recycling system in India in responding to the sustainability paradigm: an explorative study

**DOI:** 10.1038/s41598-023-30964-7

**Published:** 2023-03-13

**Authors:** Altaf Hossain Molla, Hilal Shams, Zambri Harun, Ahmad Nizam Che Kasim, Manoj Kumar Nallapaneni, Mohd Nizam Ab Rahman

**Affiliations:** 1 Department of Mechanical and Manufacturing Engineering, Faculty of Engineering and Built Environment, Universiti Kebangsaan Malaysia (UKM), 43600 Bangi, Selangor Malaysia; 2 Faculty of Economics and Management, Centre for Value Creation and Human Well-Being Studies, Universiti Kebangsaan Malaysia (UKM), 43600 Bangi, Selangor Malaysia; 3 Faculty of Business and Communication, Universiti Malaysia Perlis (UniMAP), Jalan Alor Setar-Kangar, 01000 Kangar, Perlis Malaysia; 4 School of Energy and Environment, City University of Hong Kong, Kowloon, Hong Kong, China; 5 Center for Resource Recovery, HICCER—Hariterde International Council of Circular Economy Research, Palakkad, Kerala 678631 India; 6 Center for Digital Circular Economy, HICCER—Hariterde International Council of Circular Economy Research, Palakkad, Kerala 678631 India; 7 Swiss School of Business and Management Geneva, Avenue des Morgines 12, 1213 Genève, Switzerland

**Keywords:** Climate-change mitigation, Energy infrastructure, Mechanical engineering

## Abstract

The growing number of end-of-life vehicles (ELVs) engenders a genuine concern for achieving sustainable development. Properly recycling ELV is paramount to checking pollution, reducing landfills, and conserving natural resources. The present study evaluates the sustainability of India's ELV recycling system from techno-socio-economic and environmental aspects as an instrumental step for assessing performance and progress. This investigation has performed the Strength-Weakness-Opportunity-Threat (SWOT) analysis to evaluate ELV recycling in the long-term viability and examine the critical factors and potential. This research makes practical recommendations for effectively encountering persistent challenges in the ELV recycling system based on Indian values. This research adopts an explorative and Integrated bottom-up mixed approach; it interfaces qualitative and quantitative data and secondary research. This study reveals that the social, economic, technological, and environmental aspects of the sustainability of India's ELV recycling system are comparatively limited. The SWOT analysis demonstrates that potential market size and resource recovery are more significant strengths, whereas lack of an appropriate framework and limited technology are major challenges in the recycling of ELVs in India. Sustainable development and economic viability have emerged as great opportunities, while informality and environmental impact have surfaced as primary potential threats to ELV recycling in India. This paper offers insights and yields critical real-world data that may assist in rational decision-making and developing and implementing any subsequent framework.

## Introduction

As a consequence of rapid globalization and tremendous industrialization, the number of vehicles is proliferating on the road exponentially in recent times^1,2^. After a specific period, the vehicle becomes unfit for the road and generates scrap automobiles proportionally^3,4^. The vehicles that have been retired from service and are no longer in use, end-of-life vehicles (ELVs), ELVs impose numerous threats in several aspects of society; hence proper handling of ELVs is of paramount importance^5,6^. ELV recycling has received enormous momentum. It offers excellent potential to extract as much value from ELVs and sustainable hazardous waste disposal^7–9^. Proper and efficient ELV recycling enables the recovery of a significant quantity of different materials, including Iron, Aluminium, Lead, Copper, Glass, and several rare materials^10–12^. Therefore, ELV recycling is regarded as a secondary source of materials that can preserve the finite natural resources in this world^13,14^. Besides the secondary source of materials, proper ELV recycling disposes of harmful and toxic materials in ELVs in an environmentally sound way that can reduce pollution and landfills^15–17^.

**Figure 1:**
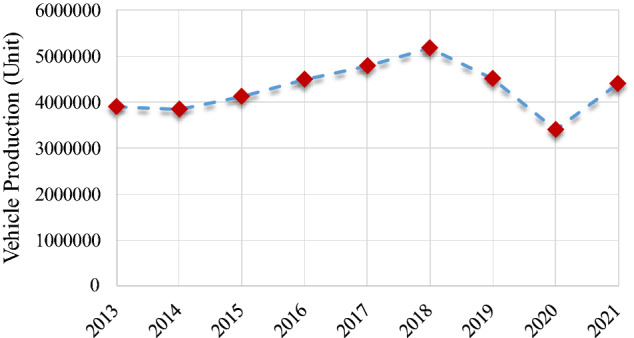
Automobile production per year in India from 2013 to 2021 (OICA^18^).

The burgeoning automobile industry in India manufactures and yields a growing number of vehicles to society every year. Figure 1 demonstrates the annual production of automobiles by the Indian automobile sector from 2013 to 2021^18^. The phenomenal expansion of India's automotive industry requires an enormous amount of distinct, precious, and scarce materials to expedite the ongoing development^19,20^. The dramatic decline of primary resources causes struggles to maintain a steady supply of raw materials for the automobile industry^21^. As ELVs incorporate a plethora of distinct ranges of materials, the recovery of materials from ELVs can assist significantly in maintaining the steady supply of materials to the material-thirsty Indian automobile sector^22–24^.

The recycling of ELVs in India is at the nascent stage and is highly chaotic and poorly managed because of the absence of legal and technical frameworks^25^. Consequently, informality in this sector prevails, which causes the bleeding of resources to waste and aggravates environmental quality^26^. The effective and practical legal and technical frameworks for ELV recycling can expedite the value extraction from ELVs in environmentally friendly ways. Countries like Japan, the USA, the EU, and China, are toiling to establish a sustainable ELV recycling system and have enacted practical regulations to govern the ELV recycling system^27–30^. As a result, they have evinced significant advancement in establishing a sustainable ELV recycling system with an excellent material recovery and recycling rate because they adopt sophisticated technology to enhance material extraction rates^31^. The material recycling and recovery rates are limited in the sector of ELV recycling in India; ELV recycling in India can only recycle certain parts, including engines, transmission systems, chassis, batteries, gearboxes, tires, rims, axles and few other economically viable body parts^32^. A significant value is squandering landfills because of the absence of advanced and cutting-edge technologies. Additionally, the de-pollution process of ELV does not adhere to environmentally friendly standards, which contributes substantially to environmental degradation^25^. The stringent legal framework and practical guidelines can enhance materials recovery rates and reduce potential greenhouse gas emissions, not to mention road and vehicle safety.

Little research sheds light on ELV recycling in India; it necessitates more explorative analysis to develop a thorough understanding of current ELV recycling practices from techno-socio-economic and environmental aspects. The absence of real-world data for the authority is stymieing rational decision-making and developing and implementing any subsequent regulatory and legal framework that succinctly embraces the techno-socio-economic and environmental aspects; it warrants more in-depth investigation on real ground. Identifying and assessing the critical factors and potential of the recycling of ELVs in India is crucial for providing future directives toward sustainability in ELV recycling; the lack of investigation in this facet demands deeper insights. The literature analysis, field investigation, and stakeholder interaction reveal that the absence of a practical framework thwarts India's ELV recycling sustainability^33^. The inadequacy of insightful recommendations that steer stakeholders toward a greater recycling rate and authority to implement legal and regulatory frameworks hinders the sustainability of ELV recycling. This issue prompts a thorough investigation.

The mentioned research inadequacies and knowledge deficiencies regarding the recycling of ELVs have inspired the current investigation. The present study evaluates the sustainability of India's ELV recycling system from techno-socio-economic and environmental aspects as a significant step to advancing understanding of the current practice and evaluating performance, and assessing progress. It offers insights and yields critical real-world data that may assist the authority in rational decision-making and developing and implementing any subsequent regulatory and legal framework. This investigation has performed the Strength-Weakness-Opportunity-Threat (SWOT) analysis to evaluate ELV recycling in the long-term viability and assess the critical factors and potential. This study's findings may assist India's authority in introducing an appropriate regulatory framework and strategic decision-making and might have significant social influence in Indian society.

### ELV recycling

**Figure 2:**
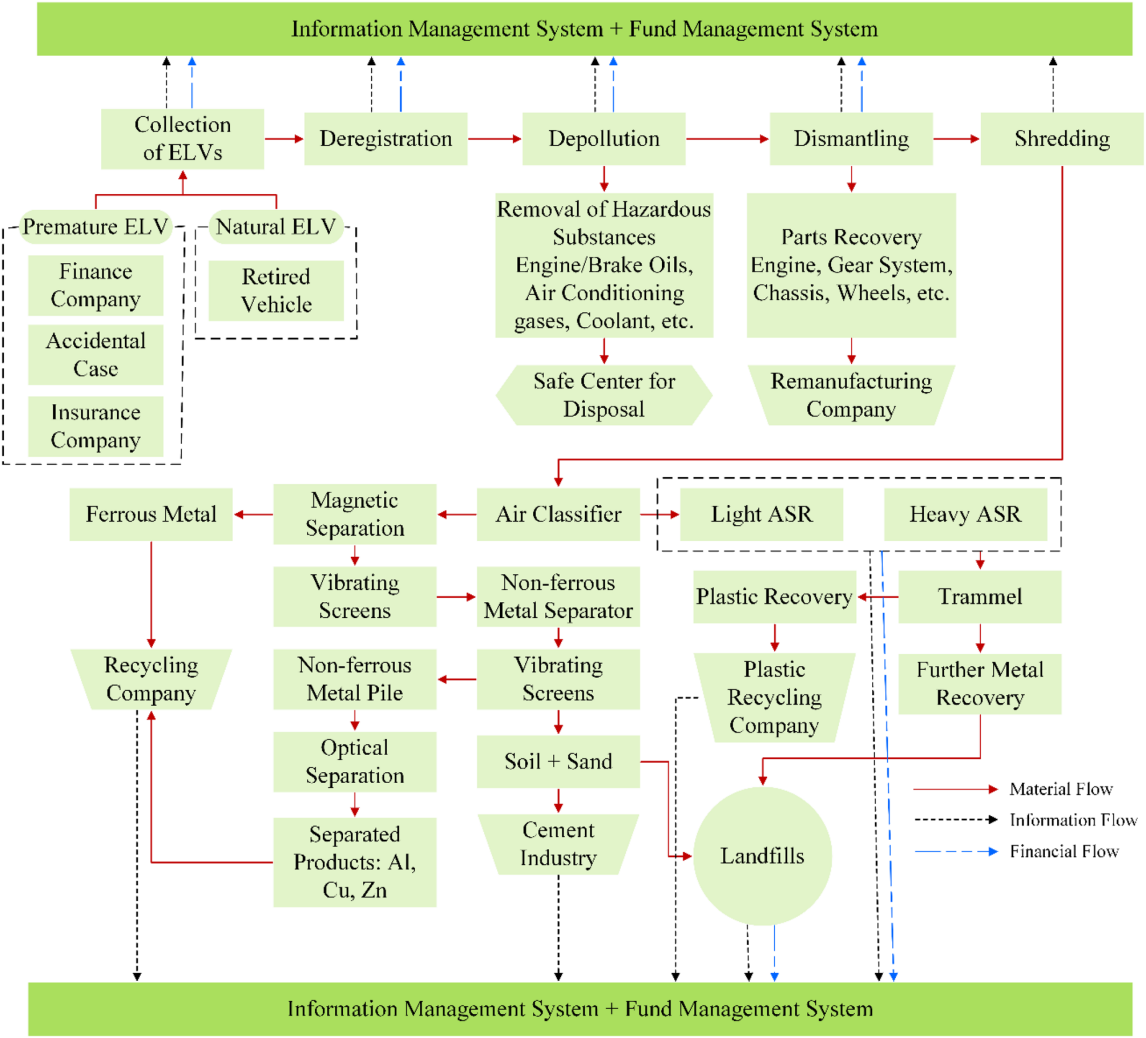
Proposed framework for ELV recycling. Adopted and redrawn from authors own source^25^.

ELV recycling system is very intricate that involves numerous complicated processes beginning from collecting ELVs and ending with automotive shredder residue (ASR) treatment^34,35^. Figure 2 demonstrates a practical framework for ELV recycling^25^. After collecting ELVs, legal paperwork needs to complete for recording details as evidence. After that, ELVs undergo depollutioning operation; in this stage, several pollutants and harmful substances, including engine oil, brake oil, coolant, and air conditioning gases, are disposed of in an environmentally sound way. In the next step, dismantling the operation, numerous recyclable and reusable parts are recovered from ELVs, such as the engine, gear system, chassis, and wheels; maximum material recovery from ELVs occurs in this stage^36^. Consequently, the remaining car hulk undergoes shredding operation for further value recovery. The shredding operation leaves automotive shredder residue that is metal-rich and contains a high calorific value^37^. Therefore, proper treatment of ASR is instrumental for higher material recovery and recycling rates^38^. Figure 2 elucidates different steps of ASR treatment for further material recovery. After further value recovery from ASR, a portion remains untreated as complete recycling of ELVs is practically not feasible; this portion disposes of at landfills. Many developed countries, like Japan and the EU, are employing sophisticated and modern technology for higher value recovery from ASR and reducing landfills as much as possible^39^.

### ELV recycling in India

**Figure 3:**
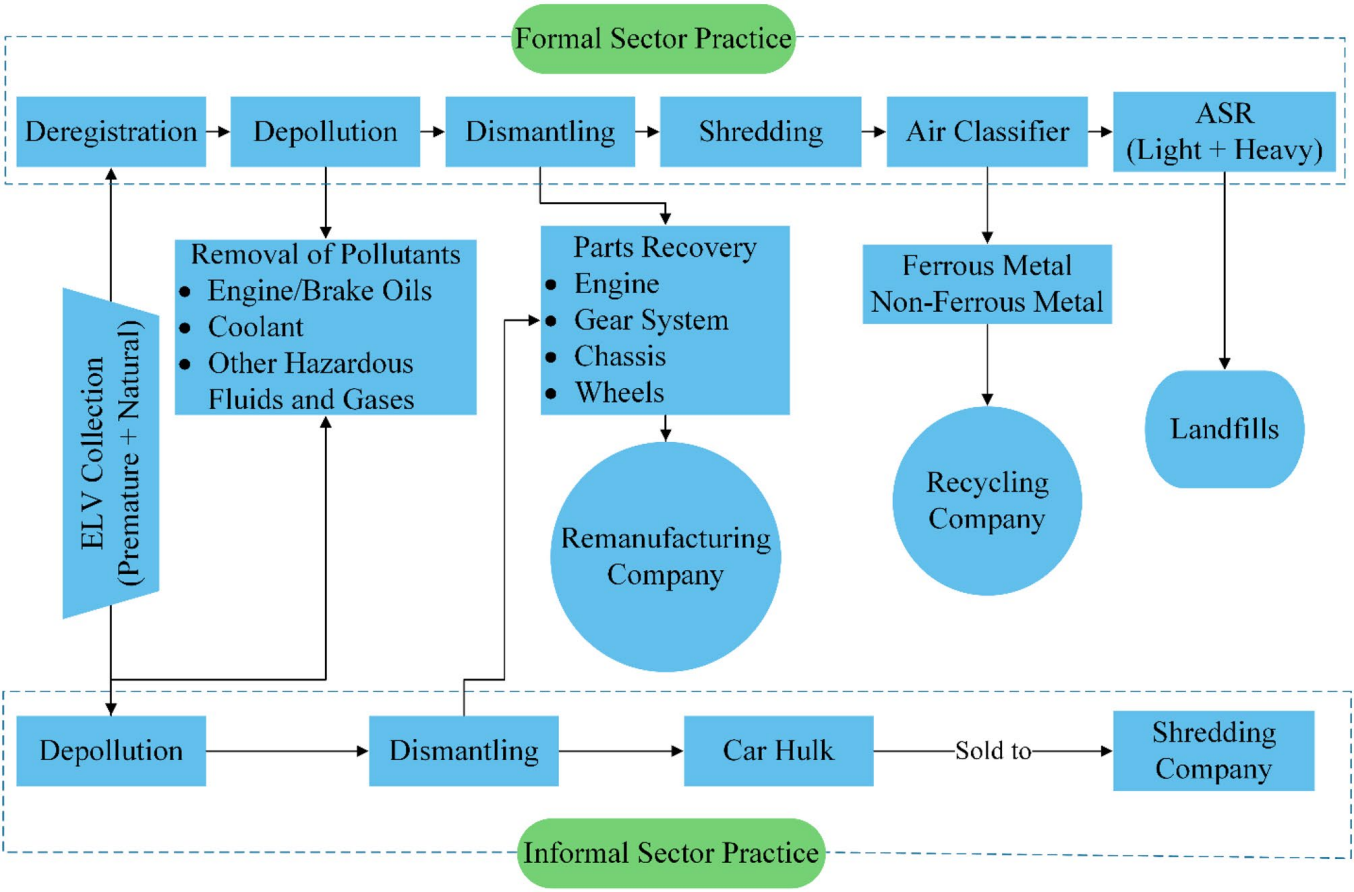
The present ELV recycling practices, including formal and informal sectors in India. Adopted and redrawn from authors own source^25^.

In India, the proper treatment of ELVs is a critical issue because ELV recycling is still in the nascent phases and is very disorganized and poorly managed^40^. Figure 3 illustrates the ELV recycling practice in India, both formal and informal sectors. The informal sector has dominated the ELV recycling sector in India since the beginning, while an insignificant number of formal ELV recycling facility centers have been emerging recently^25^. As the informal sector is solely driven by economic gain, the operations for value recovery from ELVs disregard the environmental impact, which aggravates environmental quality. In the informal sector, after perfunctory depollutioning operation, through dismantling, certain specific parts are recovered from ELVs as the value recovery; after that remaining car hulk is sold to the shredding company for further value recovery as the informal sector lacks modern and sophisticated equipment and techniques, it leads the draining of resources to waste. Whereas the formal ELV recycling facility centers are well-organized and equipped with modern and sophisticated equipment and techniques compared to the informal sector, even though the number of formal ELV recycling facility centers is insignificant. In the formal ELV recycling facility center, the operations for value recovery from ELVs adhere to standard guidelines; after dismantling operation for parts recovery from ELVs, ELVs undergo the shredding process for further value recovery, which leaves ASR, ASR is metal-rich and carry a high calorific value, partial ASR treatment is carried out for further value recovery; however, the landfill is relatively high compared to developed nations.

## Materials and methods

This research explores India's ELV recycling system by adopting an explorative and integrated bottom-up mixed approach; It interfaces qualitative and quantitative data and secondary research.

## Research design and sampling

This investigation shined the spotlight on critical automotive industrial zones in India to evaluate the sustainability of India's ELV recycling system from techno-socio-economic and environmental aspects and to perform the SWOT analysis to evaluate the ELV recycling system in the long-term viability and assess the critical factors and potential. This research developed a structured questionnaire to perform the objective evaluations that it sought. This questionnaire was evaluated and endorsed by experts and had three sections, demographic information, sustainability evaluation from techno-socio-economic and environmental aspects, and SWOT analysis. The demographic part of the questionnaire sought basic information about survey respondents, and Table 1 enumerates the demographic data and detailed information for the survey sample. This research interacted with 263 selective individuals encompassing individuals in general, vehicle owners, key stakeholders, experts, academicians, and government-authorized agencies with the structured questionnaire. This Research adopts a stratified and systematic sampling technique for determining a sample of concerned individuals. The selected respondents were informed of the study's objectives as well as instructions for completing the questionnaire. This study conducted the survey for objective measurements between September 2021 and February 2022. This investigation employed a five-point Likert scale, ranging from 1 to 5, for the data collection for this study, where '1' is "very poor", '2' is "poor", '3' is "average", '4' is "good", and '5' is "excellent". For sustainability evaluation from techno-socio-economic and environmental aspects, the ratings reflect current practices and circumstances, social and environmental awareness, stakeholder perceptions, technological development, and economic viability toward achieving sustainability. A higher rating indicates a better circumstance and applicability towards sustainability, whereas a lower rating equates with a worse situation and applicability toward achieving sustainability. This study also adopted the five-point Likert scale for the SWOT analysis, ranging from 1 to 5, where '1' is "lowest extent", and '5' is "greatest extent". The ratings for SWOT analysis factors reflect the importance as well as the significance of the factors, which allows prioritizing the factors; for strengths and weaknesses in the SWOT analysis, a rating indicates how important and significant a strength or weakness is for India's ELV recycling system; A higher rating for strengths or weaknesses in the SWOT analysis implies that the feature is more significant. For opportunities and threats in the SWOT analysis, a rating indicates the extent or degree of an opportunity or threat to India's ELV recycling system. A higher grading for opportunities or threats indicates more excellent opportunities or greater threats.

**Table 1:** Demographic information of the respondents.

Demographic factors	Classification	Frequency	Percentages (%)
Gender	Male	208	79.08
	Female	55	20.91
Age	25–30	31	11.78
	30–35	57	21.67
	35–40	71	26.99
	40–50	55	20.91
	> 50 years old	49	18.63
Education level	Undergraduate	95	36.12
	Postgraduate	123	46.76
	PhD	19	7.22
	Others	26	9.88
Income (INR)	25–50 K	51	19.39
	50–100 K	75	28.51
	100–150 K	66	25.04
	150–200 K	40	15.20
	> 200 K	31	11.78
Occupation	Manager	45	17.11
	Public service	29	11.02
	Academician	35	13.30
	Automobile Industrialist	68	25.85
	Social worker	61	23.19
	Others	25	9.5

### Instruments development

This investigation employed a quantitative research method to perform objective measurements through a structured questionnaire. Secondary research was an integral part of the methodology of this study. This investigation conducted secondary research to explore and analyze the present level of information about ELV recycling, develop comprehensive insights, gain detailed information, and evaluate the research limitations and gaps in the ELV recycling system in India. The literature review enabled the development of the robust foundation of this research. It significantly assisted in factors selected for evaluating sustainability from techno-socio-economic and environmental aspects and the SWOT analysis and making practical recommendations based on Indian values to resolve the persistent issues to expedite the sustainability of the ELV recycling system in India. The questionnaire items were drawn from literature reviews. Initially, more items were selected from the literature to conduct this study. Eventually, after a thorough review to assess the importance, relevance, and significance of the factors, a few factors were dropped from the questionnaire to make the questionnaire more precise, pertinent, and applicable; experts advised and recommended this exclusion. Furthermore, the relevancy, applicability, and subjectivity of all selected factors were checked and verified by the ELV stakeholders consultation. Social awareness and understanding of ELV recycling, current practices in society, and government-sponsored awareness-raising campaigns were considered for selecting factors for assessing sustainability from the social dimension. Satisfaction with the received monetary value for ELV, economic gain of stakeholders, expansion of the sector, investment for advanced types of equipment for energy-efficient operations, and financial assistance from authority determined the factors for evaluating sustainability from the economic dimension. The factors for assessing sustainability from the technological aspect were derived from equipment upgradation, application of energy-efficient equipment, recycling capacity, and facility of glass and plastic recycling and ASR treatment. The factors for evaluating sustainability from the environmental dimension were decided based on the amount of landfill, ecological degradation, energy consumption, carbon emission, and hazardous waste management.

For the SWOT analysis factors selection, for strengths, India's internal resources and tangible assets, distinctive qualities, and technological development were taken into consideration; for weaknesses, the lacking qualities, resource limitations, and malpractices were taken into account; for opportunities, emerging economy, investment, future goals, and initiatives were considered; for threats, potential vulnerabilities, legislation, environmental quality, and globalization were taken into account.

Table 2 illustrates the final questionnaire items for evaluating sustainability from techno-socio-economic and environmental aspects. Table 3 enumerates the final questionnaire items for performing the SWOT analysis. The questionnaire included 24 items for assessing sustainability from techno-socio-economic and environmental aspects, namely, the social aspects (SOA) (8 items), the economic aspects (ECA) (6 items), the technological aspects (TEA) (5 items), and the environmental aspects (ENA) (5 items). In addition, the questionnaire also incorporated another 20 items for the SWOT analysis, namely, the strengths (5 items), the weaknesses (5 items), the opportunities (5 items), and the threats (5 items). Besides the quantitative research, this investigation performed qualitative research to holistically explore India's ELV recycling system. The qualitative research method interfaced with individual in-depth interviews and field investigation. The individual in-depth interviews unveiled real-world situations naturalistically. Field inspections and observations yielded a comprehensive understanding and holistic view of current practices. Interviews encompass perspectives and live experiences of all stratums of participants. These insights were imperative for the SWOT analysis, developing a profound understanding of India's ELV recycling system and making practical recommendations based on Indian values.

**Table 2:** The questionnaire items for evaluating sustainability from techno-socio-economic and environmental aspects.

Dimension	Item	Description	References
Social aspects	SOA1	Knowledge and understanding of ELV recycling	^3,41–43^
	SOA2	Awareness of pollution caused by ELVs	
	SOA3	Awareness of benefits from ELV recycling	
	SOA4	Understanding of the ELV's propensity for road accident	
	SOA5	Support the ELV recycling	
	SOA6	The practice of ELV recycling in the neighborhood	
	SOA7	Access to ELV recycling center	
	SOA8	The support from the government in promoting awareness of ELV recycling	
Economic aspects	ECA1	Satisfy with the received monetary value for ELV	^4,44–47^
	ECA2	Expansion of the ELV recycling sector	
	ECA3	Increase profit from the ELV recycling sector compared to previous years	
	ECA4	Increase investment for enhancing material recovery and recycling rates	
	ECA5	Increase investment in energy-efficient operations	
	ECA6	Receive financial assistance from the authority	
Technological aspects	TEA1	Upgrade the types of equipment for enhancing the recycling rate from ELVs	^8,20,37,48^
	TEA2	Introduce energy-efficient equipment	
	TEA3	Capacity for recycling all types of vehicles, including two-wheeler, three-wheeler, private vehicles, commercial passenger and goods vehicles, military vehicles, and others	
	TEA4	Facility for recycling glass and plastic components	
	TEA5	ASR treatment facility	
Environmental aspects	ENA1	Awareness about pollution for improper recycling	^5–7,49–52^
	ENA2	Reduction in landfills	
	ENA3	Reduction in energy consumption	
	ENA4	Minimizing carbon emissions from recycling operations	
	ENA5	Comply with proper guidelines for disposing of hazardous waste

**Table 3:** The questionnaire items for the SWOT analysis.

	Parameter	Factor	References
SWOT analysis	Strengths	Potential market size	^1,25,26,41,53–56^
		Resource recovery	
		Increasing demand	
		Waste management system	
		Skilled and cheaper labor	
	Weaknesses	Lack of appropriate framework	
		Limited technology	
		Lack of knowledge and awareness	
		Environmental degradation and pollution	
		Vehicle deregistration and ELV collection	
	Opportunities	Towards circular economy and sustainable development	
		Economic viability	
		Employment creation	
		Resource efficiency	
		Energy efficiency	
	Threats	Informality in ELV recycling sector	
		Environmental impacts of ELV recycling	
		Different value chain	
		Lax regulation and policy	
		Market barrier	

### Data analysis

**Figure 4:**
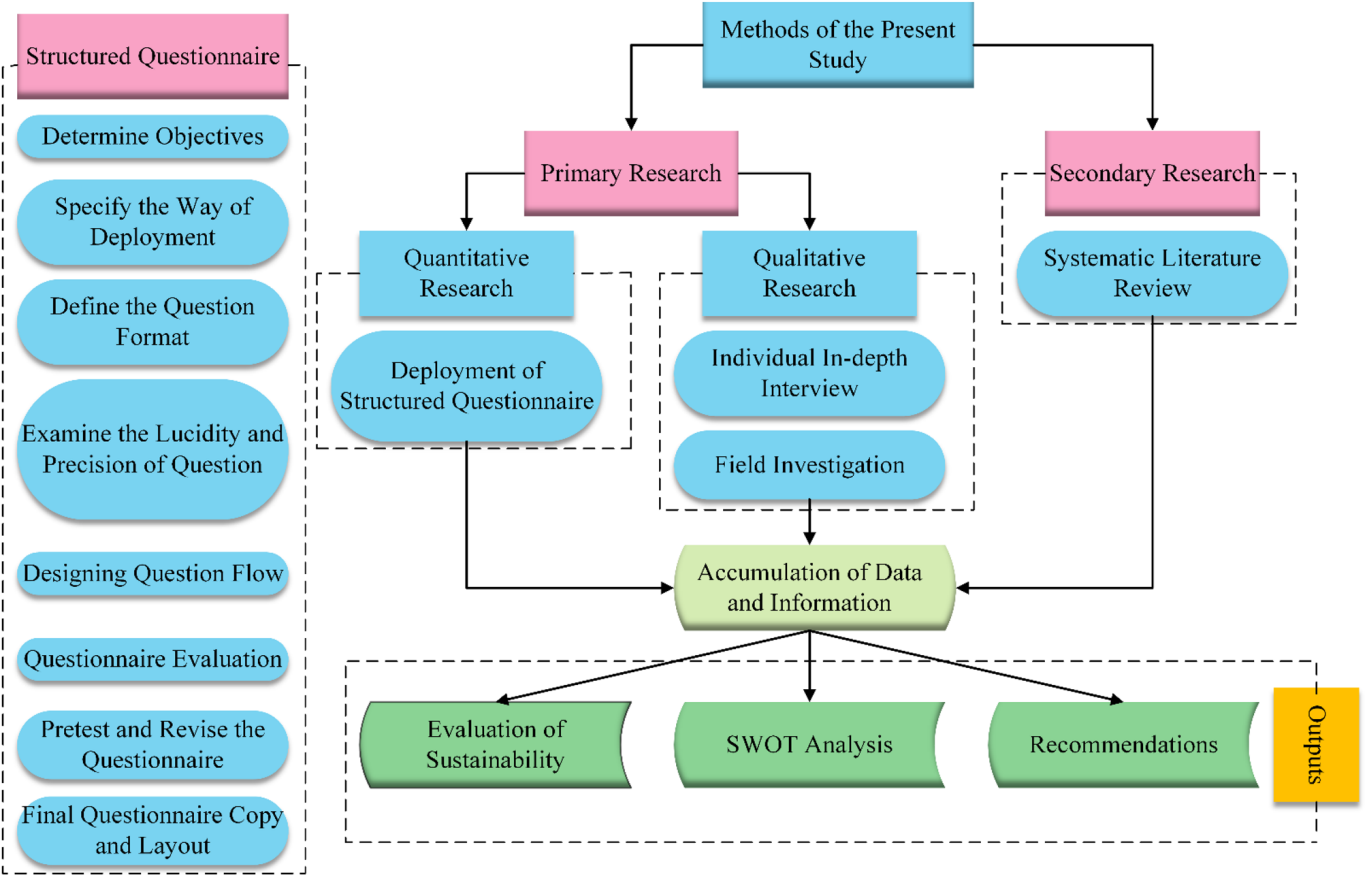
Methodology integrating structured questionnaire, primary research, and secondary research.

Data analysis is a significant phase that entails interpreting and analyzing the obtained quantitative as well as qualitative data. All the data collected for objective measurement for the present study was based on the five-point Likert scale. Microsoft Excel and SPSS software programs were employed to analyze the quantitative data, whereas ATLAS and Nvivo software programs were used to interpret and rationalize the qualitative data. To evaluate the sustainability of India's ELV recycling system from techno-socio-economic and environmental aspects, this study calculated the mean value of collected data and arranged the items in the social, economic, technological, and environmental aspects in an ascending way for visualization. For the SWOT analysis, the mean value of the parameters in the SWOT analysis was calculated and arranged the parameters in an ascending way for visualization. Figure 4 depicts the general methodology of this study.

## Critical findings

As this research evaluates the sustainability of India's ELV recycling system from techno-socio-economic and environmental aspects by performing objective measurements, this section delineates the critical findings of this study.

### Social aspects

**Figure 5:**
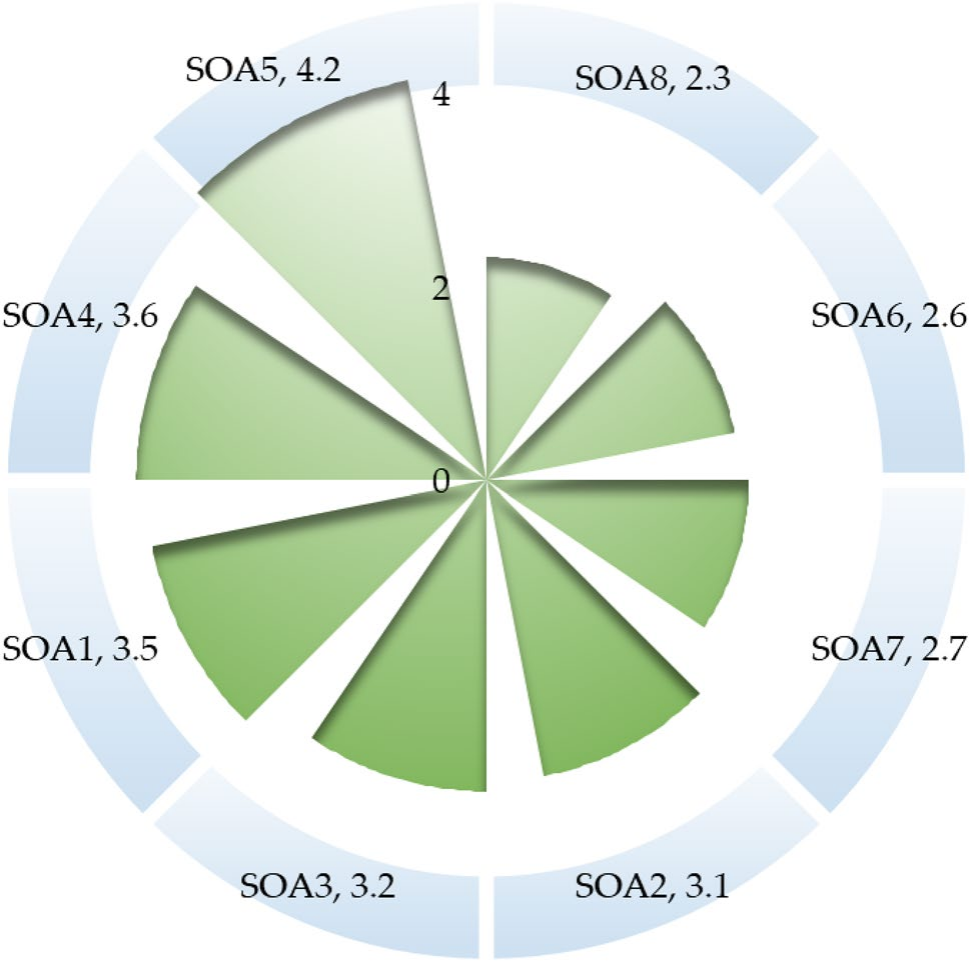
Sustainability evaluation of ELV recycling system from social dimensions.

Social awareness is of paramount importance for establishing an effective and sustainable ELV recycling system, as it encourages individuals to recycle their abandoned vehicles. A greater awareness level in society is more conducive to adopting and implementing any subsequent ELV recycling framework^41,43^. Figure 5 delineates the sustainability evaluation from social aspects. The knowledge and understanding regarding ELV recycling (SOA1) are mediocre in society; a comprehensive understanding of ELV recycling is instrumental for implementing and designing an effective ELV recycling system. Awareness of the environmental impact caused by ELVs (SOA2) and knowledge of the benefits of ELVs recycling (SOA3) are moderate in the community. The awareness of the advantages of ELV recycling is very crucial as it inspires individuals to recycle the ELVs; a higher awareness indicates a greater acceptability of ELV recycling. This study unveils that understanding the ELV's propensity for road accidents (SOA4) is acceptable; 3.6 out of 5 scales. This research reveals that the community has high inspiration and support for ELV recycling (SOA5), 4.2 out of 5 scales. This high inspiration and support will expedite the development of ELV recycling and assist in implementing and adapting an effective framework for ELV recycling. Even though individuals hold a high drive and motive for ELV recycling, the practice of ELV recycling in the community (SOA6) is still limited, 2.6 out of 5 scales. This investigation demonstrates that access to the ELV recycling center (SOA7) for ELV recycling is also limited, 2.7 out of 5 scales. The ELV recycling system is in a nascent stage in India; individuals still rely on local mechanics to sell their abandoned vehicles in return for insignificant monetary value, as very few formal ELV recycling facility center exists in the community. Awareness plays a vital role in adapting ELV recycling; however, the support from the government in promoting awareness of ELV recycling (SOA8) is insufficient and poor, 2.3 out of 5 scales. The role of authority is paramount in designing an effective ELV recycling system and making relevant policies, regulations, and guidelines. The Indian authority should promote ELV recycling to enhance awareness and understanding in society.

### Economic aspects

**Figure 6:**
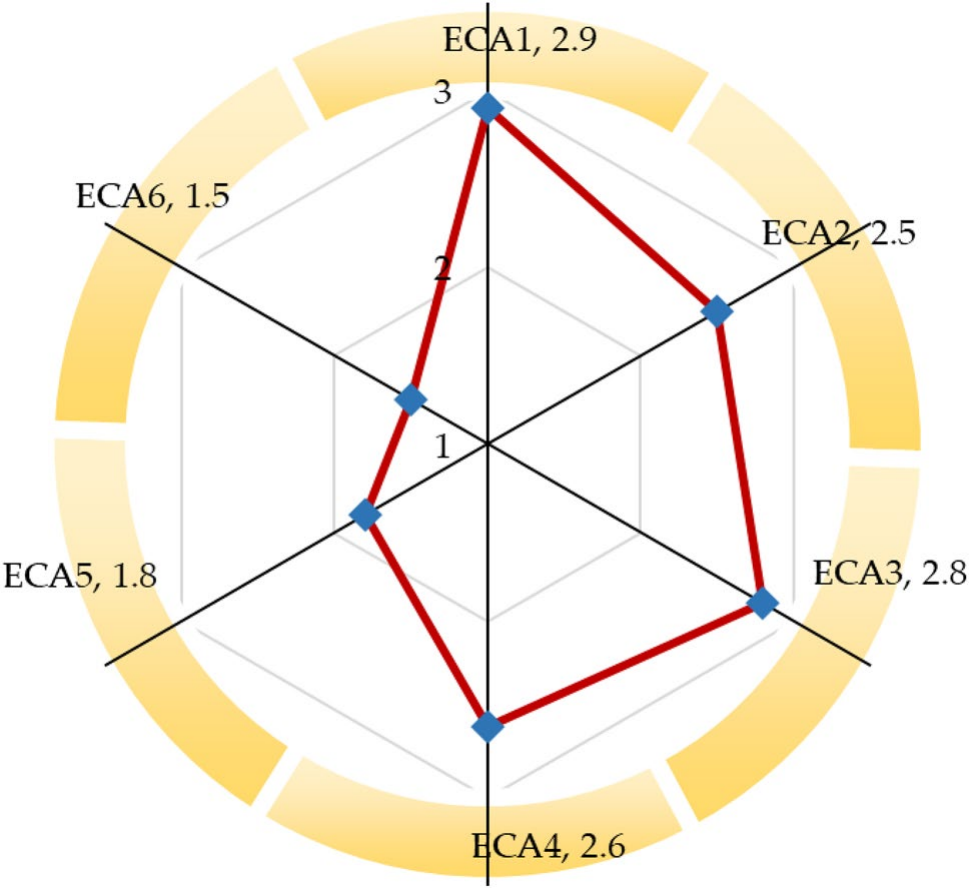
Sustainability evaluation of ELV recycling system from economic dimensions.

Economic gain is a great motivator; receiving appropriate monetary values for ELV inspires individuals to recycle their scrap vehicles. Conversely, making more profit from ELV recycling prompts stakeholders to invest in energy-efficient operations and higher material recovery from ELV^7,14^. Figure 6 demonstrates the sustainability evaluation from economic dimensions. This study reveals that the monetary value received for the abandoned vehicle (ECA1) is inadequate, 2.9 out of 5 scales. It indicates that individuals receive substandard monetary value for their abandoned vehicles, which can prevent them from recycling their abandoned vehicles. This research finds that the expansion of the ELV recycling sector (ECA2) has been limited in recent years, 2.5 out of 5 scales. It suggests that the ELV recycling business is not expanding much. The authority should assist in expanding the formal ELV recycling sector for long-term viability and sustainability. An increase in economic gain prompts the sector to expand; this study demonstrates that the profit made from the ELV recycling sector compared to previous years (ECA3) is moderate, 2.8 out of 5 scales. It indicates ELV recycling is still one of the most profitable business sectors. Limited resource recovery is a persistent issue in India's ELV recycling sector; this investigation reveals that the investment made by ELV recyclers for enhancing material recovery and recycling rates from ELVs (ECA4) is still adequate, 2.6 out of 5 scales. It suggests that the ELV recycling sector is still operating with traditional technology. The ELV recycling sector requires sophisticated technology to enhance materials recycling and recovery rates. The world is facing an energy crisis, and this issue is becoming more severe; energy-efficient operations in the industry can lead to significant energy savings and an increase in economic gain^57^. This research unveils that investment in energy-efficient operations in the ELV recycling sector (ECA5) is poor, 1.8 out of 5 scales. It implies that ELV recyclers have not yet upgraded their equipment. Incentives and financial assistance authorities are conducive to developing a sustainable industrial sector; this exploration demonstrates that the ELV recycling sector receives inadequate financial assistance from the authority (ECA6), 1.5 out of 5 scales. It suggests that the ELV recycling sector is running without sufficient incentives and assistance from the authority.

### Technological aspects

**Figure 7:**
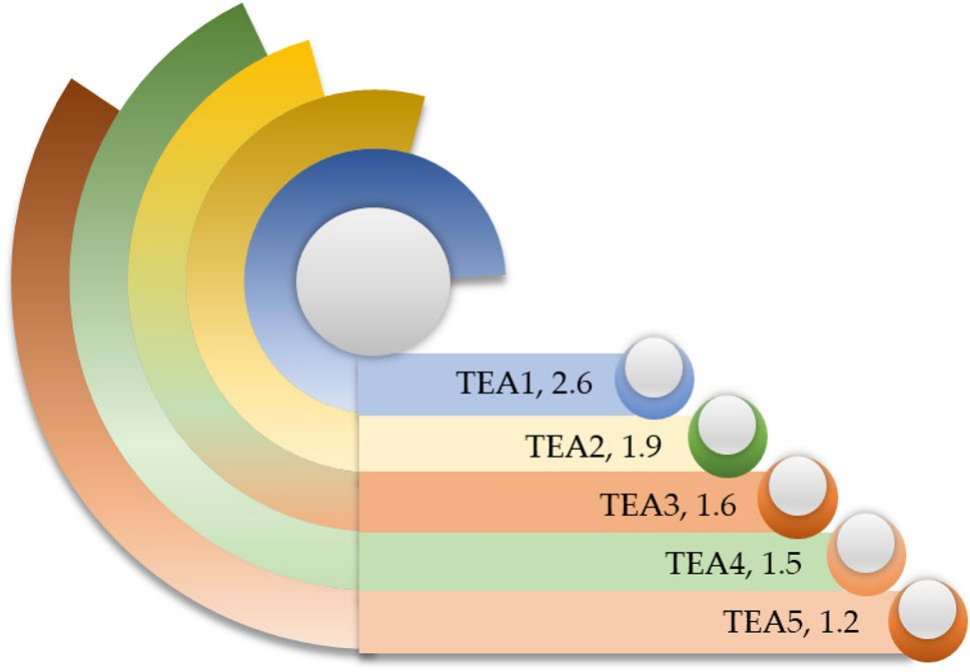
Sustainability evaluation of ELV recycling system from technological dimensions.

Advanced and sophisticated technology is instrumental, as it enables higher material recovery and recycling rates, lower energy consumption, and reduces the emission of greenhouse gases^5,13,51,58^. Figure 7 delineates the evaluation of sustainability from technological dimensions. This investigation finds that the upgradation of the types of equipment for enhancing the recycling rate from ELVs (TEA1) is limited, 2.6 out of 5 scales. It suggests that most ELV recycling sector operates with outdated technology for value recovery from ELVs. As the ELV recycling sector operates with conventional machinery, it consumes a lot of energy; this research reveals that introducing energy-efficient equipment in the ELV recycling sector (TEA2) is limited, 1.9 out of 5 scales. Energy-efficient equipment contributes to reducing the emission of greenhouse gases; hence, higher energy consumption indicates a greater amount of greenhouse gases emission^59^. The informal sector dominates India's ELV recycling sector, and a recycling center is incapable of recycling all kinds of vehicles; this study demonstrates that the capacity for recycling all types of vehicles, including two-wheeler, three-wheeler, private vehicles, commercial passenger and goods vehicles, military vehicles, and others (TEA3) is poor, 1.6 out of 5 scales. Glass and plastic are the significant part of the vehicle, which can be recycled^60,61^. This exploration exhibits that the capability for recycling glass and plastic components (TEA4) is very limited, 1.5 out of 5 scales. It implies that material recovery and recycling rates can be elevated by emphasizing glass and plastic components recycling. ASR is a metal-rich mixture that contains a high value; through effective ASR treatment, significant further value can be recovered^37^. This study delineates that the ASR treatment facility in India's ELV recycling sector (TEA5) is scarce, 1.2 out of 5 scales. It indicates that most of the ELV recycling sector in India operates without an ASR treatment facility.

### Environmental aspects

**Figure 8:**
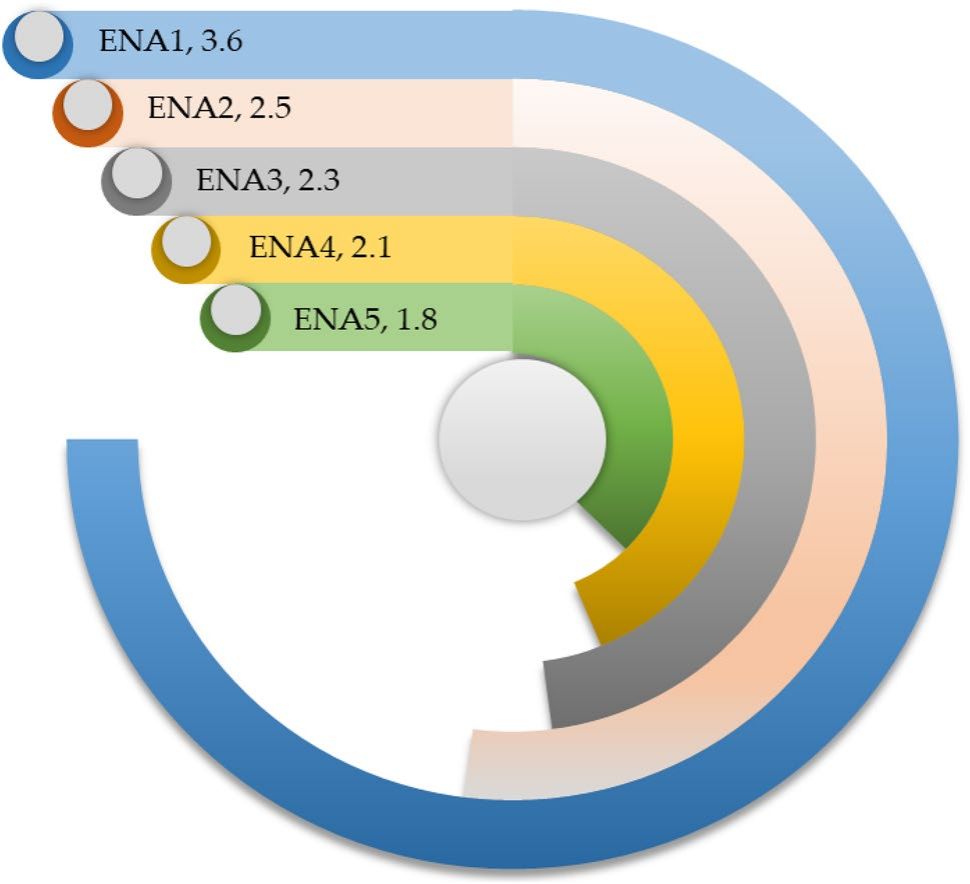
Sustainability evaluation of ELV recycling system from environmental dimensions.

ELV contains several hazardous and harmful substances, and proper disposal of these toxic substances is imperative to safeguard the environment^7,49,62^. Figure 8 illustrates the evaluation of sustainability from environmental dimensions. This exploration reveals that the awareness of pollution caused by improper recycling (ENA1) is acceptable, 3.6 out of 5 scales. Improper ELV recycling causes environmental degradation^53^; this awareness of pollution caused by improper recycling is conducive to adopting an efficient ELV recycling framework. As mentioned, India's ELV recycling system operates with relatively lower materials recycling and recovery rates; therefore, landfill is relatively higher. This investigation unveils that the reduction in landfills (ENA2) is limited, 2.5 out of 5 scales. It indicates that still further value can be recovered by reducing landfills. Higher energy consumption is one of the persistent issues in industries; higher energy consumption degenerates the environment. This research demonstrates that the effort to reduce energy consumption (ENA3) is poor, 2.3 out of 5 scales. The reduction in carbon emissions is a global challenge; the United Nations (UN) has taken numerous initiatives to reduce carbon emissions. This study delineates that the endeavor to minimize carbon emissions from recycling operations (ENA4) is poor, 2.1 out of 5 scales. ELVs encompass several detrimental and hazardous substances, and sustainable disposal of these harmful materials and waste is paramount^49^. This exploration finds that following proper guidelines for disposing of hazardous waste (ENA5) is scarce in India's ELV recycling sector, 1.8 out of 5 scales. It indicates that the generated harmful waste is not disposed of in an environmentally sound way, which is a potential environmental threat.

## SWOT analysis

This research has performed the SWOT analysis to evaluate the competitiveness of India's ELV recycling system by assessing critical factors as well as present and future potential. The SWOT analysis investigates strengths, weaknesses, opportunities, and threats in India's ELV recycling system, yielding a fact-based analysis, fresh perspectives, and profound insights.

### Strengths

**Figure 9:**
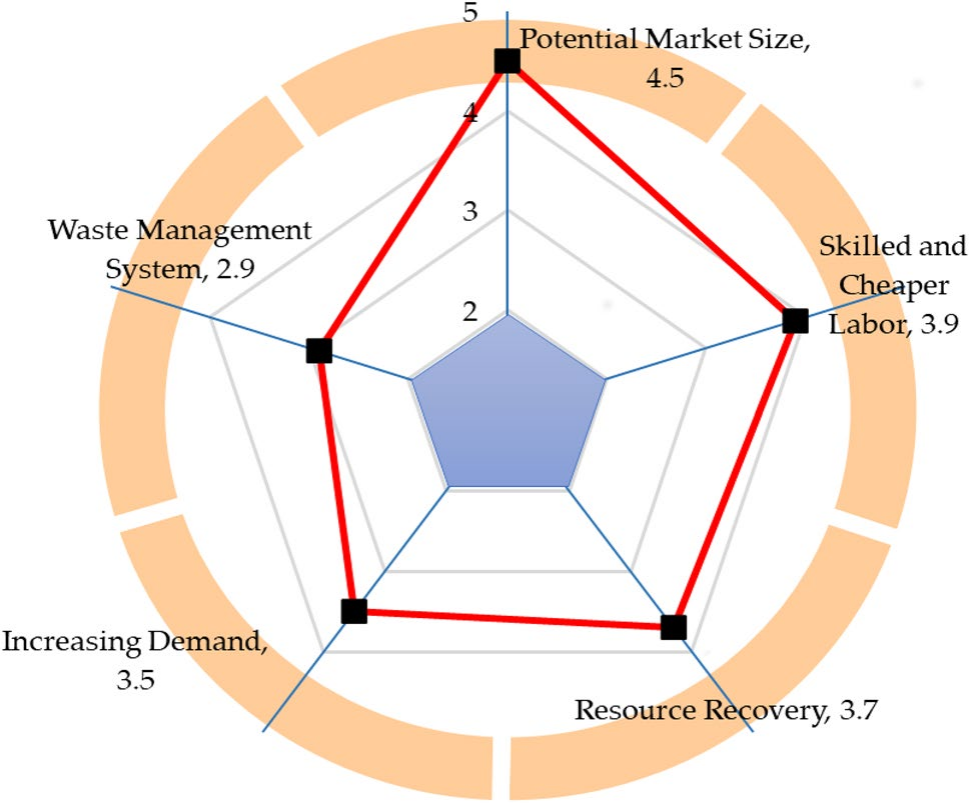
Strengths of India's ELV recycling system.

Figure 9 demonstrates the strengths of India's ELV recycling system. The potential market size has emerged as India's most significant strength of the ELV recycling system, with a rating of 4.5 out of 5 scales. India's automotive industry has been dramatically expanding in the last decades; consequently, phenomenal development draws foreign investment. That makes India one of the largest automobile manufacturers in the world. India generates the highest number of engineers and technicians that assists the automotive industry in snowballing continuously. The ELV recycling sector can leverage the most prominent automobile industry, as ELV stakeholders can sell recovered recyclable parts from ELVs to car manufacturers. That assists in creating a homogeneous value chain. The enormous potential market size is a great potency for the ELV recycling sector. Skilled and cheaper labor has surfaced as the second most significant strength of India's ELV recycling sector, with a rating of 3.9 out of 5 scales. India has become a great source of skilled workers and technicians. The availability of skilled and cheap labor is an excellent leverage for India's ELV recycling system. Resource recovery has appeared as one of the substantial strengths, with a rating of 3.7 out of 5 scales. ELV recycling offers the recovery of a host of distinct and scarce materials and components as ELVs encompass various materials. Indian authority struggles to meet the demand for raw or virgin materials in automotive industries to continue expanding. Effective ELV recycling may serve as a secondary source of materials. The potency of recovering materials and components from ELV is a significant potential that ELV recycling offers. Increasing demand has been identified as a significant strength of India's ELV recycling sector, with a rating of 3.5 out of 5 scales. Industrialization and modernization, accompanied by rising working and middle-class incomes, stoke car demand. The growing population in India contributes substantially to car demand, and, in addition, India's exports are increasing rapidly. Domestic and export car demand can benefit the ELV recycling sector to flourish. The waste management system has been established as a substantial strength for ELV recycling, with a rating of 2.9 out of 5 scales. India has been developing and designing an effective waste management system; therefore, the waste management system can assist in developing an effective ELV recycling system.

### Weaknesses

**Figure 10:**
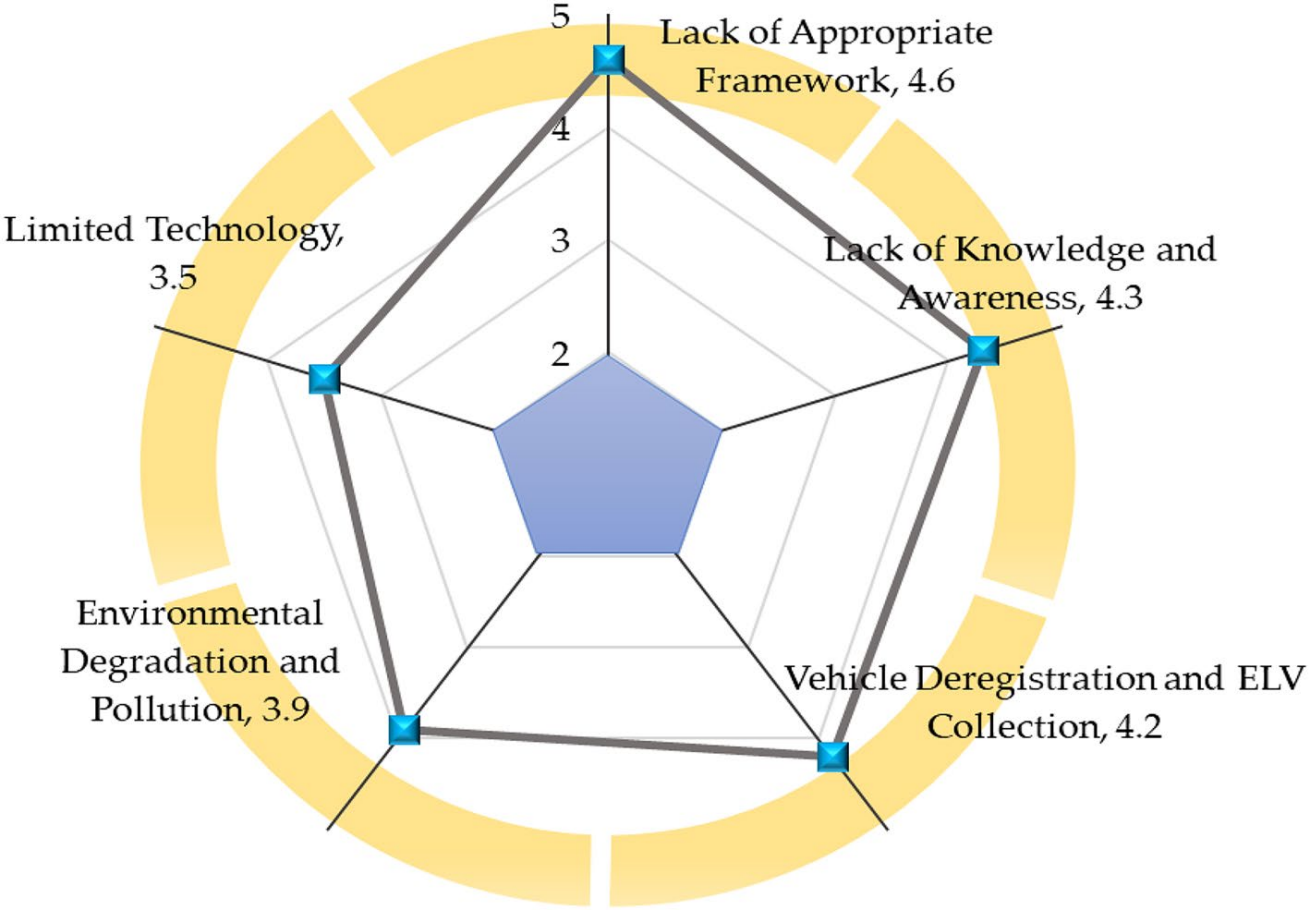
Weaknesses of India's ELV recycling system.

Figure 10 delineates the weaknesses of India's ELV recycling system. The SWOT analysis unveils that the lack of an appropriate framework has emerged as the most severe and critical weakness of India's ELV recycling system, with a rating of 4.6 out of 5 scales. India's ELV recycling system is predominant in the informal sector. India lacks a comprehensive and appropriate framework for ELV recycling, which leads to a governance vacuum. The lack of a proper framework thwarts the development of India's ELV recycling sector. India's ELV recycling requires a practical framework. The lack of knowledge and awareness has surfaced as the second most severe and critical issue in ELV recycling in India, with a rating of 4.3 out of 5 scales. Social awareness is a crucial factor in ELV recycling. The lack of awareness is evident among locals of India. Indians are unconcerned about environmental degradation. In addition, the Indian government is reluctant to promote public awareness and education. Vehicle deregistration and ELV collection have appeared as one of the significant challenging issues in ELV recycling, with a rating of 4.2 out of 5 scales. The collection of ELVs and the deregistration process are the initial steps of ELV recycling; Indian authorities have yet to design and implement the vehicle deregistration process and ELVs collection process. Environmental degradation and pollution have been identified as critical issues in ELV recycling in India, with a rating of 3.9 out of 5 scales. The ELV recycling sector disposes of hazardous waste, not in an environmentally sound way, which degenerates environmental quality. Limited technology has been regarded as a severe weakness of ELV recycling, with a rating of 3.5 out of 5 scales. Poor technology is a persistent issue in India's ELV recycling system. Most ELV dismantlers have rudimentary tools for ELV dismantling operations, which significantly affects the materials recovery, recycling, and extraction rate.

### Opportunities

**Figure 11:**
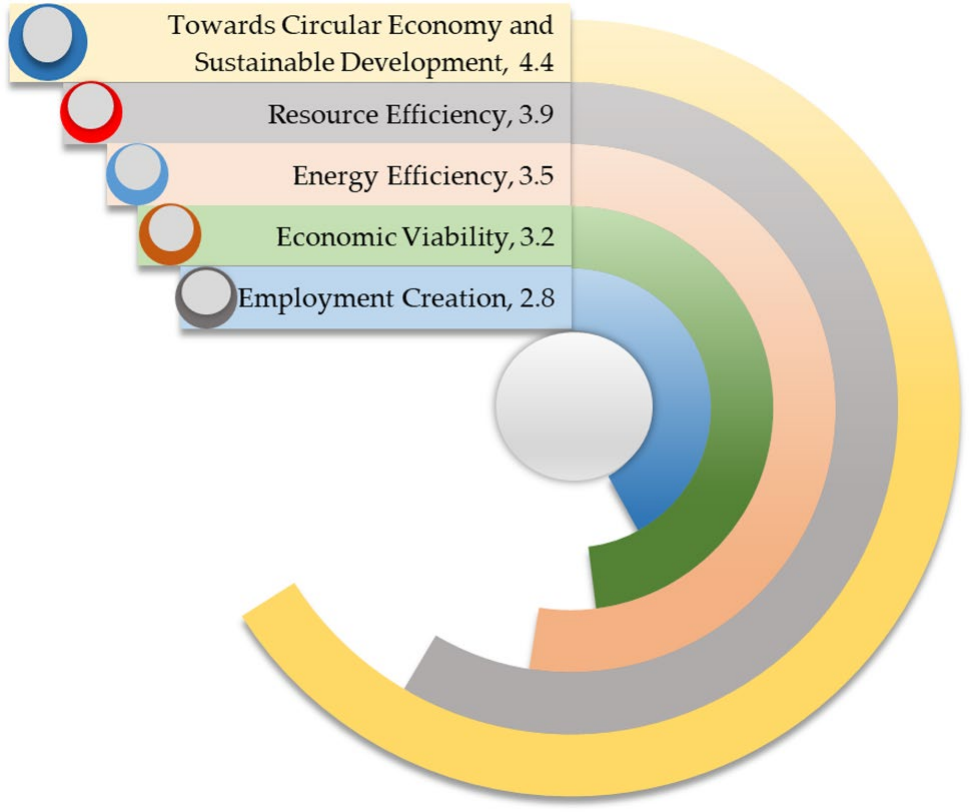
Opportunities for India's ELV recycling system.

Figure 11 illustrates the opportunities of India's ELV recycling system. The SWOT analysis reveals that the opportunity for the circular economy and sustainable development has surfaced as the greatest opportunity for India's ELV recycling sector, which has been rated 4.4 out of 5 scales. Effective ELV recycling yields excellent opportunities to develop a circular economy in India's automobile industry. The reuse, recycling, and recovery process extract values from ELVs in environmentally sounding ways that advance sustainability and expedite the transition toward a circular economy. ELV recycling can assist the world in achieving a sustainable and prosperous future for this world. Resource efficiency has emerged as the second greatest opportunity for ELV recycling in India, which has been rated 3.9 out of 5 scales. ELV recycling can preserve and use the finite resources of this world sustainably while respecting the environment. Energy efficiency has been identified as one of the greater opportunities for ELV recycling, with a rating of 3.5 out of 5 scales. ELV recycling promotes energy efficiency as recycling saves a lot of energy and prevents energy waste. Economic viability has appeared as a potential opportunity for ELV recycling, with a rating of 3.2 out of 5 scales. ELV recycling offers excellent economic viability and sustainability for all stakeholders involved in India's automobile industry. That creates more flexibility and adaptability for all stakeholders in ELV recycling by obtaining the best quality products, taking care of the environment and keeping the business going. Employment creation has been regarded as a potential opportunity for ELV recycling, with a rating of 2.8 out of 5 scales. The ELV recycling sectors generate employment for numerous individuals to keep the economy growing at a steady pace. In addition, this sector is receiving a lot of attention from entrepreneurs as it offers significant economic viability, flexibility, and business adaptability.

### Threats

**Figure 12:**
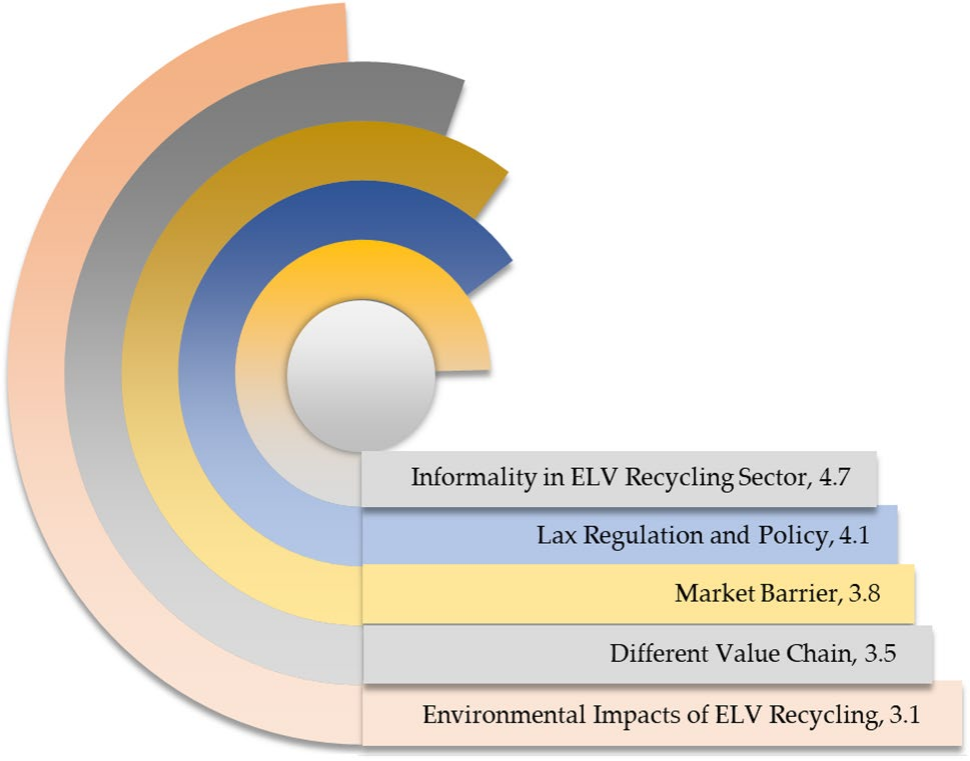
Threats to India's ELV recycling system.

Figure 12 depicts the threats to India's ELV recycling system. The SWOT analysis demonstrates that informality in the ELV recycling sector has emerged as the greatest threat to the ELV recycling system in India, with a rating of 4.7 out of 5 scales. The informal sectors dominate India's ELV recycling sector. They do not follow any guidelines for ELV dismantling operations, which poses significant environmental threats. In addition, improper handling of resources is prevalent in informal sectors as they recover only certain economically important parts from ELVs. The lax regulation and policy have surfaced as a grave threat to ELV recycling, with a rating of 4.1 out of 5 scales. India requires to enact appropriate policies to govern the ELV recycling sector. The vacuum of laws and regulations makes the ELV recycling sector disordered and chaotic. The market barrier has appeared as one of the prominent threats to the ELV recycling sector, with a rating of 3.8 out of 5 scales. The market obstacle is apparent in India's ELV recycling sector. The different value chain has been regarded as one of the grave threats to ELV recycling in India, with a rating of 3.5 out of 5 scales. Albeit the vehicle manufacturing industry and the ELV recycling sector are inextricably connected, no collaboration exists between them; they have different value chains, respectively, which thwarts sustainability in ELV recycling in India. The environmental impact of ELV recycling has been identified as a prominent threat to ELV recycling, with a rating of 3.1 out of 5 scales. ELVs contain several hazardous solid, liquid, and gaseous substances. Proper handling of these substances is paramount. In India's ELV recycling system, stakeholders generally throw these detrimental substances into landfills, which degrades the environmental quality significantly.

## Discussions

This study evaluates the sustainability of India's ELV recycling system from techno-socio-economic and environmental dimensions. This research finds that knowledge and understanding of ELV recycling are mediocre in society, but individuals express great support for ELV recycling, which is considered substantial backing for the implementation and adaptation of an effective ELV recycling system. This investigation unveils that the practice of abandoned vehicle recycling in society is limited; lack of awareness prevents individuals from properly ELV recycling their abandoned vehicles. The lack of support for enhancing public awareness from authorities has emerged in this investigation. The authority should endeavour to enhance the awareness level in society, which will drive people to recycle their abandoned vehicles. This research underlines the social aspects of the sustainability of India's ELV recycling system for elevating the lives of people in Indian society. This study might have a profound impact on people to save energy, reduce landfills, and safeguard the environment.

Moreover, the findings of this study demonstrate that the amount paid to the end-user of the vehicle for the ELV is insufficient because the monetary value of ELVs in India depends on the market price of iron only. The insufficient monetary value of ELV discourages the end-user of the vehicle from recycling vehicle. The authority should devise an appropriate legal framework to govern the ELV recycling system. This investigation finds that ELV recycling is still one of India's more profitable businesses. However, the ELV recycling sector is not growing rapidly. This research reveals that the investment for enhancing materials recovery and recycling and energy-efficient operations is limited, which leads to poor materials recovery rates and high energy consumption. It indicates that financial constraint is impeding the development of the ELV recycling sector. Lack of assistance and incentives from authorities has surfaced as a prominent issue in the ELV recycling sector. The authorities should take the initiative for financial assistance for ELV stakeholders to grow the ELV recycling sector sustainably.

Most ELV recycling centers operate with rudimentary machinery, leading to low materials recovery, a persistent issue in the ELV recycling sector. There are different types of abandoned vehicles in the community, including two-wheeler, three-wheeler, private vehicles, commercial passenger and goods vehicles, military vehicles, and others; one ELV recycling center can recycle only a specific type of vehicles, not all kinds of vehicles. The glass and plastic components can be recycled^61,63^; nonetheless, the glass and plastic components are not recycled at most of the ELV recycling centers, leading to significant value waste. ASR treatment is a crucial step for value recovery from ELVs^64^. Still, the lack of modern techniques and machinery prevents most ELV recycling centers from treating ASR, which also leads to significant value waste. Increasing landfills causes great concern in society, but very little effort has been put forward to reduce landfills. This study finds that minimal effort has been made to reduce carbon emissions and maintain the proper guidelines for disposing of hazardous wastes generated from ELV recycling. Hazardous and harmful wastes from the ELV recycling sector are required to dispose of in an environmentally friendly way^38,49^. The Indian authority should establish a safe center for hazardous waste handling to reduce the substantial environmental threat. The energy used in the ELV recycling sector, and its supportive and dependent industries comes from finite non-renewable energy sources, which have several inevitable implications for the environment and public health, including greenhouse gas emissions and air and land pollution. Renewable energy can play a crucial role as it offers a low-emission, clean and infinite energy source. The application of renewable energy can reduce carbon emissions from the ELV recycling sector significantly and facilitate the achievement of sustainability^65^.

In addition, this research has performed the SWOT analysis to evaluate ELV recycling in the long-term viability and assess the critical factors and potential. The SWOT analysis demonstrates that the potential market size and skilled and cheap labor are the superior strengths of India's ELV recycling sector, while the lack of appropriate framework, knowledge and awareness are more severe weaknesses of the ELV recycling system. The combination of the growing automobile market and cheap and skilled labor draws significant foreign investments that keep India's automobile sector expanding constantly. Resource recovery, growing demands, and waste management system initiatives are significant strengths that can improve the performance of the ELV recycling system in India. The lack of an appropriate framework makes India's ELV recycling chaotic and disorganized; the authority should develop an appropriate framework to assist the ELV stakeholders in advancing and providing standard guidelines. Besides the lack of an appropriate framework, the authority should address the issues, including lack of awareness and knowledge, vehicle deregistration and ELV collection, limited technology, and pollution, to establish an efficient ELV recycling system.

Towards circular economy and sustainable development and economic viability have emerged as the greater opportunities for ELV recycling, whereas informality in the ELV recycling sector and market barriers have appeared as the greater threats to the ELV recycling system. Establishing an efficient ELV recycling system can create significant opportunities, including developing a circular economy, achieving sustainable development goals, resource recovery, reduction in energy consumption, economic prosperity and job creation. Informality, absence of policy and regulation, and different value chains are palpable potential threats to India's ELV recycling system; these threats can thwart the achievement of sustainability in the ELV recycling sector. The value chain in the automotive industry and the ELV recycling sector should be interconnected; nonetheless, no collaboration exists between vehicle manufacturers and the stakeholders involved in ELV recycling; lack of cooperation prevents information flow^66^. As a result, stakeholders engaged in ELV recycling cannot recycle modern vehicles with conventional technology until car manufacturers share their contemporary technologies, which leaves numerous vehicles untreated and make ELV recycling arduous and inefficient. The findings of the SWOT analysis might have significant practical impacts on society as it emphasizes maximizing strengths while reducing weaknesses and benefits from opportunities, and eventually, it underscores the potential threats to the ELV recycling sector. The SWOT analysis findings may enable the current ELV recycling system to be more productive and improve the decision-making process.

This study sheds light on India's ELV recycling system and yields critical and insightful interpretations of factors contributing to sustainability. The findings of this investigation reflect the ground-level practice of ELV recycling; hence this study's outcomes may assist the Indian authority in the appropriate decision-making and implementing and designing policy, regulation and framework for ELV recycling. As this research evaluates sustainability from techno-socio-economic and environmental dimensions by underscoring group-level practices, therefore outcomes of this investigation may assist in subsequent research. This research may facilitate the authority to transform the informality-dominated India's ELV recycling sector towards a more sustainable ELV recycling system.

## Recommendations and future research prospects

This study makes practical recommendations as the reflection of insights gained from observation and analysis have been performed in the previous section.

### Development of practical framework

Designing a practical framework is imperative to achieving sustainable goals. Informality-dominated India's ELV recycling sector unveils the absence of practical frameworks. By developing practical frameworks, Indian authorities can provide directives for achieving sustainability in the ELV recycling sector by enhancing material recovery, recycling, and extraction rates while respecting the environment. In addition, providing practical frameworks to stakeholders will assist the government in governing the ELV recycling sector systemically. As practical frameworks offer resolutions to numerous persistent issues in India's ELV recycling system, authorities should urgently design appropriate guidelines and provide them to stakeholders.

### Promote public awareness

Public awareness plays a critical role in sustainability in the ELV recycling system. In India, public awareness is relatively low, which impedes ELV recycling significantly. Increasing public awareness level is a demanding task. The authority in India should immediately take appropriate actions to enhance awareness levels. The government can tailor awareness-raising endeavours through a systematic training program, education, reaching targeted audiences, and awareness-raising activities.

### Development of fund management

The government should develop a mutual fund immediately with the collaboration of car manufacturers, stakeholders, and car owners, as the ELV recycling system is associated with several expenses, such as recycling fees, the cost of awareness-raising initiatives, financial assistance, and informal stakeholders, subsides and waste management. This fund ought to be used for promoting sustainability and green technology.

### Establishment of information sharing center

The lack of stakeholder cooperation is palpable in India's ELV recycling system. The Indian government should immediately establish an information-sharing center to collect and share the required information with all stakeholders. The information-sharing center will make ELV dismantling operations easy and more effective.

### Hazardous waste management and decarbonization

ELVs encompass several hazardous substances, including poisonous electronic materials, waste liquids and oils, automotive shredder residue, and other detrimental residues, that should be disposed of in environmentally sound ways. Hazardous waste management will deal with harmful wastes and dispose of these wastes in sustainable practices. India's 2070 decarbonization goal is a significant and ambitious target, requiring a combination of government policies and framework for stakeholders, private sector initiatives, and societal awareness to achieve. An appropriate waste management system can significantly contribute to a decarbonization plan by reducing greenhouse gas emissions, promoting sustainable land use, and promoting a circular economy. By implementing measures such as composting, recycling, waste-to-energy, and sustainable land use, a waste management system can help reduce carbon emissions and promote a more sustainable future. The Indian government should urgently form a hazardous waste management board as the quality of the environment is deteriorating dramatically in India.

### Performance assessment

Performance evaluation plays an imperative role in enhancing product quality, standardizing ELV operations, safeguarding the environment, and promoting sustainable development and green technology. Currently, there is no performance evaluation facility in India. The Indian government should immediately design a performance evaluation system to evaluate the progress towards sustainability.

### Future research prospects

The present study has evaluated the sustainability of India's ELV recycling system from techno-socio-economic and environmental dimensions and performed the SWOT analysis based on ground-level practice. Based on this research and the research gap in the literature review, subsequent research can be conducted. Structural equation modeling (SEM) can be performed to investigate the impacts of social, economic, technological, and environmental aspects on sustainability, respectively, which requires further in-depth investigation. The fourth industrial revolution (IR 4.0) emphasizes automation, the internet of things (IoT), artificial intelligence (AI), smart factory, digital business models, and digitization and integration of vertical and horizontal value chains. The drivers and barriers to adopting and implementing IR 4.0 in ELV recycling, automobile, and manufacturing industries in India, require further explorations. This exploration will reveal the readiness for IR 4.0. The development of ELV recycling has been impeded by the absence of a practical framework; an effective framework equipped with the features mentioned in IR 4.0, blockchain^67^, and circular economy business model^68^ principles is required to support the development of ELV recycling in India, which prompts in-depth investigation Environmental degradation has become a significant challenge in India, and the evaluation of emissions from ELV recycling sectors and air quality in the vicinity of the ELV recycling sector needs further investigation. ASR is a metal-rich mixture and is not treated for value recovery in the ELV recycling sector in India because of the lack of technique and machinery. An in-depth investigation is required for ASR treatment. Stakeholders are the key players in establishing an effective ELV recycling system in India; the lack of collaboration between stakeholders is evident, which impedes material flow and information flow, which requires in-depth investigation. Economic gain inspires stakeholders to invest in energy-efficient operations and higher material recycling and recovery; material recycling and recovery rates are limited; hence the study of the economic feasibility of ELV recycling in India prompts a deeper investigation. As India's environmental quality is degenerating continuously, hence the study of the impact of ELV recycling on environmental and public health necessitates further research. Also, in recent years, India started witnessing an electric vehicle (EV) boom; this would create an entirely different set of ELVs (i.e., EV-based ELVs), and the sustainable transition could depend on various other conditions. Some evidence from European Union suggested that EVs sustainable transition can't be achieved unless conditions like the use of renewable sources, local development, and battery recycling are met^69^. This might be true in the case of India too, so detailed further research focusing on India's EV-based ELVs could be considered. Localizing ELV recycling facilities in remote and sub-urban areas in India has advantages and disadvantages; this depends on the specific circumstances and perspectives. While it may reduce the potential negative impacts on public health and the environment, it may also increase transportation emissions and face infrastructure limitations. It is essential to carefully evaluate the benefits and drawbacks of locating ELV recycling facilities in remote areas, considering factors such as public health, environmental impact, economic development, and sustainable infrastructure. This issue requires a thorough investigation before moving forward with the localization of ELV recycling to especially remote areas and sub-urban regions in India.

## Conclusions and limitations

This research has evaluated the sustainability of India's ELV recycling system from techno-socio-economic and environmental aspects as an instrumental step for assessing performance and progress. The findings of this paper unveil that the knowledge and understanding of ELV recycling are moderate; in contrast, individuals show significant support for ELV recycling. The practice of abandoned car recycling is limited in society. ELV recycling is still one of the more profitable businesses but growing gradually. Investment in enhancing material recovery and energy saving is limited. The ELV recycling sector in India operates with conventional equipment and machinery, leading to limited material recovery rates from ELVs. Increasing landfills is a great challenge for authority. The generated harmful waste from ELV recycling is not disposed of in an environmentally sound way, which is a potential environmental threat. This study has performed the SWOT analysis to evaluate the competitiveness of India's ELV recycling system and yield a fact-based analysis and fresh perspectives. Potential market size, economic viability, and increasing demand are favourable factors; on the contrary, lack of appropriate framework, limited technology, and informality in the ELV recycling sector are critical concerns in India's ELV recycling sector. This investigation makes practical recommendations for effectively encountering persistent challenges in the ELV recycling system, enhancing materials recovery, recycling, and extraction rates, and expediting sustainable development. This research yields critical real-world data that might be instrumental for rational decision-making and developing and implementing any subsequent regulatory and legal framework that succinctly embraces the techno-socio-economic and environmental aspects. This study may assist India's government in enacting the following policy for achieving sustainable goals in ELV recycling.

To the authors' best knowledge, this research represents the initial study for evaluating the sustainability of India's ELV recycling system from techno-socio-economic and environmental aspects and performing the weighted SWOT analysis to emphasize the most critical factors and prioritize the elements according to their significance and importance to assess the competitiveness of India's ELV recycling system and to overcome the limitations of the simple SWOT analysis. Consequently, this paper will assist in the future advanced study of India's ELV recycling.

We also highlight a few potential drawbacks of this research study. This research interviewed 263 selective individuals to evaluate the sustainability of India's ELV recycling system from techno-socio-economic and environmental aspects and perform the SWOT analysis; the greater sample size might have given more precise outcomes. Secondary research is crucial for providing the ground for the research. This research has performed objective measurements through predefined items. The lack of contemporary literature reviews about India's ELV recycling system made the items selection for evaluating sustainability from techno-socio-economic and environmental aspects and performing the SWOT analysis very difficult. India nurtures great diversity in the world; different states have different cultures, languages, inspirations, and economic conditions; it is a cumbersome task to divide the sample size equally among people of different cultures. This study focused on the significant automobile hubs in India only. As mentioned in this paper, India's ELV recycling sector is dominated by the informal sector, and informal ELV recyclers are very suspicious of disclosing information about their practices to researchers because of fear of legal proceedings. It was an arduous task to obtain information about their perspectives and practices.

## Data Availability

All data generated or analyzed during this study are included in this published article. Also, they are available from the corresponding author upon request.

## Abbreviations

ELV: End-of-life vehicle
SWOT: Strength-weakness-opportunity-threat
ASR: Automotive shredder residue
SOA: Social aspect
ECA: Economic aspect
TEA: Technological aspect
ENA: Environmental aspect
SEM: Structural equation modeling
IoT: Internet of things
IR 4.0: The fourth industrial revolution
AI: Artificial intelligence
EV: Electric vehicle
